# IPOM versus eTEP as minimally invasive approaches for ventral/incisional hernias: a systematic review and meta-analysis

**DOI:** 10.1007/s10029-025-03319-6

**Published:** 2025-04-14

**Authors:** Yeong Huei Desmond Chuah, Angus Lloyd, Shaheel Mohammad Sahebally

**Affiliations:** 1 Department of General and Colorectal Surgery, Tallaght University Hospital, Dublin, D24 NR0A Ireland; 2 School of Medicine, Trinity College Dublin, Dublin, Ireland

**Keywords:** Minimally invasive, Abdominal wall hernia, Ventral, IPOM, ETEP

## Abstract

**Purpose:**

Enhanced-view totally extraperitoneal (eTEP) repair of ventral hernias is an emerging modality that places synthetic mesh in the retrorectus space and obviates its fixation. We aimed to compare outcomes between eTEP and minimally invasive intraperitoneal onlay mesh (IPOM) repair techniques.

**Methods:**

A PRISMA-compliant meta-analysis searching PubMed, EMBASE and CENTRAL databases from January 2010 till August 2024, was performed. All studies comparing IPOM versus eTEP were included. The primary objective was postoperative pain at day 7 (POD7) whereas secondary objectives included operative time, length of stay (LOS), intraoperative and postoperative complications and recurrence. Random effects models were used to calculate pooled effect size estimates. Sensitivity analyses were also performed.

**Results:**

Twelve studies (3 randomized, 9 observational) capturing 868 patients (452 IPOM, 416 eTEP) were included. Most hernias were primary ventral (n = 806). Most studies (10/12) adopted a laparoscopic approach whilst two employed robotic techniques. IPOM was associated with significantly higher pain scores at POD7 (VAS; visual analog scale; MD 3.01, 95%CI = 1.28–4.75, p = 0.0007), longer LOS (MD 0.65 days, 95%CI = 0.27–1.04, p = 0.001) but shorter operative time (MD - 53.69 min, 95%CI = - 69.65- - 37.73, p < 0.00001). However, there was no differences in intraoperative (OR 2.04, 95%CI = 0.81–5.17, p = 0.13), postoperative (OR 1.15, 95%CI = 0.54–2.46, p = 0.72) complications or recurrence (OR 2.08, 95%CI = 0.79–5.46, p = 0.14). On sensitivity analyses, comparing laparoscopic IPOM with defect closure (IPOM +) versus eTEP, similar results prevailed.

**Conclusions:**

IPOM(+) is associated with more postoperative pain at one week and a longer hospital stay. However, no differences were observed in complications or recurrence between the two techniques.

**Supplementary Information:**

The online version contains supplementary material available at 10.1007/s10029-025-03319-6.

## Introduction

Abdominal wall ventral hernias constitute a significant workload for the surgeon, with over 300,000 repaired annually in both Europe and the United States, and are responsible for an economic burden of 3.2 billion dollars each year [1]. They consist of primary (umbilical, paraumbilical, epigastric and Spigelian), incisional and parastomal hernias. Despite an improved understanding in closure techniques (i.e. optimal suture-to-wound length ratio) and biomechanics [2, 3], incisional hernias remain a common complication after abdominal surgery, with a reported incidence as high as 60% in high-risk cohorts [4, 5]. Primary ventral hernias, on the other hand, occur spontaneously and affect 20% of the healthy adult population [6]. Whilst they can simply be aesthetically displeasing, they can also cause discomfort, pain and worse still, result in incarceration of abdominal contents, necessitating emergency interventions.

Ventral hernia mesh repair techniques have rapidly evolved over the last two decades. Whilst it is generally accepted that laparoscopic repair is superior to open repair in terms of seroma formation, length of hospital stay and recurrence, it can also result in complications [7–9]. The traditional laparoscopic technique involved reduction of the hernial sac and bridging the defect with an intraperitoneal prosthesis without defect closure (IPOM) [10]. However, increased short-term postoperative pain (due to the use of tackers) [11] and early recurrence (due to mesh migration) [12] have led to the development of alternative techniques, such as enhanced-view totally extraperitoneal (eTEP) repair [13], whereby a prosthesis is laid out flat in the dissected retromuscular space, without the need for mesh fixation.

We aimed to compare short- and long-term outcomes between minimally invasive IPOM and eTEP techniques for ventral hernias in this current meta-analysis.

## Methods

This systemic review and meta-analysis was conducted in accordance with the Cochrane Handbook of Systematic Review & Meta-Analysis of interventions (v6.2) principles and reported according to the Preferred Reporting Items for Systemic Review and Meta-Analyses (PRISMA) [14] and Assessing the Methodological Quality of Systematic Reviews (AMSTAR 2) [15] guidelines. It was registered in the PROSPERO database (CRD42024534570).

### Eligibility criteria

All English language studies directly comparing minimally invasive intraperitoneal onlay mesh repair (IPOM; laparoscopic or robotic) and enhanced-view totally extraperitoneal repair (eTEP; laparoscopic or robotic) for the management of symptomatic ventral hernias (primary or incisional/recurrent) were eligible for inclusion in our meta-analysis. Studies evaluating parastomal hernia repair were excluded. In addition, studies were excluded if the retromuscular space was accessed transabdominally (i.e. transabdominal retromuscular repair; TARM) or if open abdominal wall hernia repair was carried out or if outcomes of interest were inadequately reported. Unpublished reports and the grey literature were also excluded.

### Search strategy

Medline, EMBASE and CENTRAL (Cochrane Central Register of Controlled Trials) databases as well as Google Scholar were searched from January 2010 to August 2024 for relevant studies using combinations of the following medical subject heading (MeSH) terms [“IPOM” OR “intraperitoneal onlay mesh”] AND [“eTEP” OR “extraperitoneal repair” OR “enhanced view” OR “extended view” AND [“ventral” OR “abdominal wall” OR “hernia”]. The last search was conducted on April 30 th, 2024. Two independent investigators (YHDC and AL) reviewed the titles and abstracts and full texts of potentially eligible studies were obtained. The bibliographies of the latter studies were further screened for other potential studies for inclusion. Where discrepancies existed, the opinion of the senior author (SMS) was sought.

### Study outcomes

The primary objective was pain at postoperative day 7 measured in VAS (visual analog scale). Secondary objectives included operative time (minutes), length of stay (LOS; days), intraoperative (bowel/vascular injury) and postoperative (seroma, haematoma, surgical site infection etc.) complications and recurrence. Random effects models were used to calculate pooled effect size estimates. Sensitivity analyses were also performed.

### Data collection

Data were extracted independently by YHDC and AL onto a password-protected Excel sheet. The following information was retrieved: authors’ names, journal, year of publication, gender, mean age, sample size, study design, type of minimally invasive surgery (laparoscopic or robotic), inclusion and exclusion criteria, size of defect, types of mesh used, defect closure (or not), mesh tacking (or not), use of drains, postoperative outcomes and length of follow up.

### Data analysis

The Review Manager software (RevMan v5.3. Copenhagen: The Nordic Cochrane Centre, The Cochrane Collaboration, 2012) was used for data analysis. For dichotomous variables (i.e. recurrence, complications) the odds ratio (OR) was calculated with its variance and 95% confidence interval (CI). For continuous variables (i.e. operative time, length of stay and VAS scores), the mean difference (MD) was calculated with its 95% CI. All pooled outcome measures were determined using the random effects model as described by DerSimonian and Laird.(24) The results of each outcome assessed were displayed on a forest plot with 95% CI. A *p* value < 0.05 was considered statistically significant. The existing heterogeneity between different studies was estimated by the *I*^2^ inconsistency test with values < 25% indicating low, between 25- 75% moderate and > 75% high statistical heterogeneity present. The risk of bias was assessed independently by two authors (YHDC and AL) using the ROBINS- 1 tool [16, 17] and the Cochrane risk of bias version 2 tool (RoB 2) [18, 19]. Publication bias was also assessed graphically.

## Results

### Study selection and characteristics

**Fig. 1:**
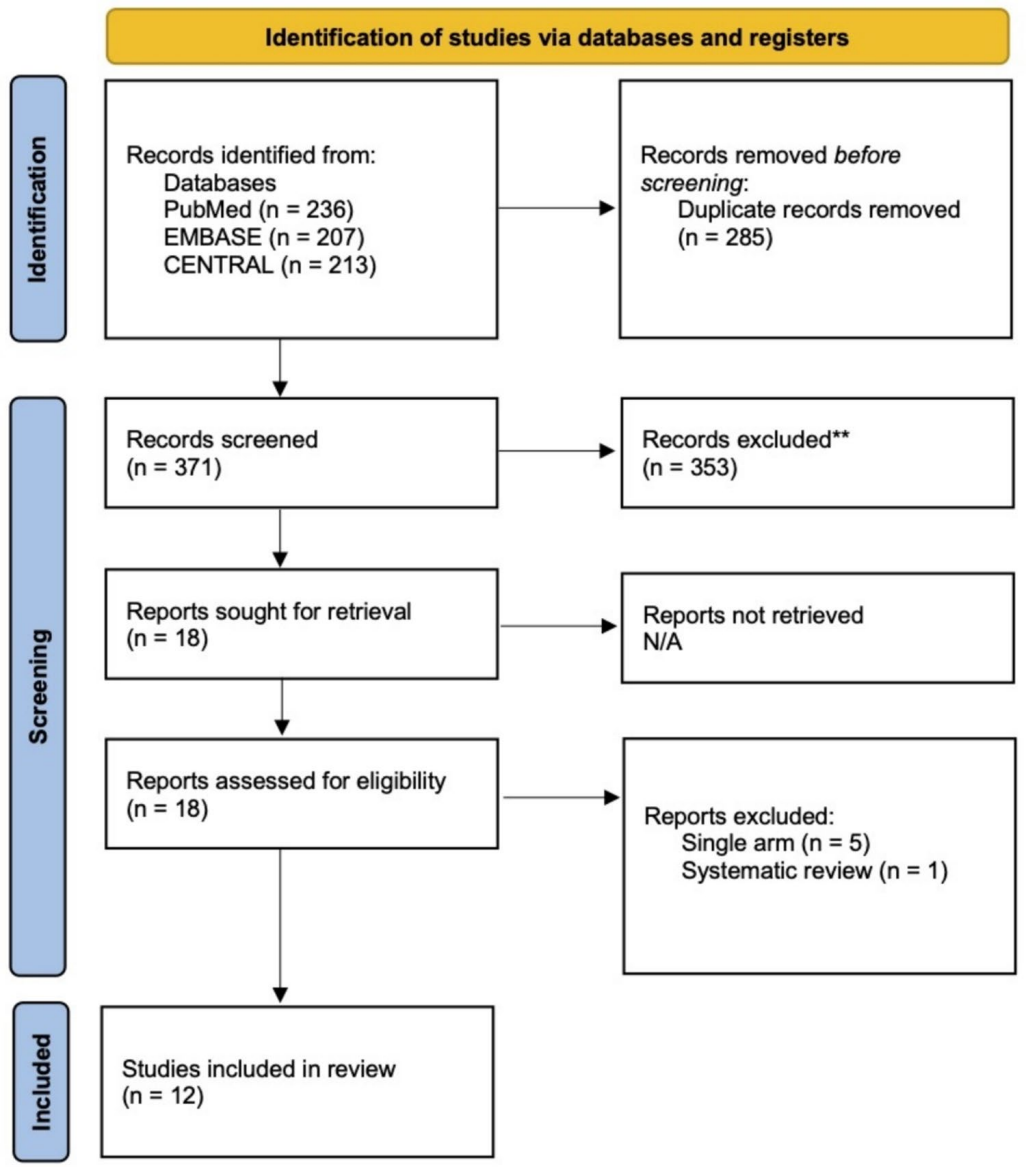
PRISMA flow chart of selection process

The initial search identified 656 articles. After application of inclusion and exclusion criteria, twelve studies [20–31] (9 observational, 3 randomized) were ultimately included in this systematic review and meta-analysis. A flow diagram highlighting the selection process is shown in Fig. 1. Six of the studies originated in Asia (4 in India [24, 26, 27, 30], 1 in Bangladesh [31], and 1 in China [20]), 4 in Europe (Spain [21], Turkey [22], Denmark [25], Bulgaria [29]), and 2 in the United States [23, 28]. Most (10 out of 12) studies adopted a laparoscopic approach whilst two employed robotic techniques [23, 28]. Further study characteristics are provided in Tables 1 and 2.

**Table 1:** Study characteristics

Study	Study design	Country	Study year	Patient numbers IPOM/eTEP	Approach
20	Retrospective	China	2023	30/22	Laparoscopic
21	Case–control	Spain	2021	39/40	Laparoscopic
22	Retrospective	Turkey	2023	44/30	Laparoscopic (+ TAR)
23	RCT	US	2023	49/51	Robotic (+ TAR)
24	RCT	India	2022	30/30	Laparoscopic (+ TAR)
25	Retrospective	Denmark	2022	43/29	Laparoscopic
26	RCT	India	2024	25/25	Laparoscopic
27	Prospective	India	2020	46/46	Laparoscopic (+ TAR)
28	Retrospective	US	2020	68/68	Robotic (+ TAR)
29	Retrospective	Bulgaria	2019	27/27	Laparoscopic
30	Prospective	India	2020	30/30	Laparoscopic
32	Prospective	Bangladesh	2022	20/18	Laparoscopic

*RCT* Randomised controlled trial, *TAR* Transversus abdominis release

**Table 2:** Baseline characteristics

Characteristics (Mean ± SD)	IPOM (n = 452)	eTEP (n = 416)	*p-value*
Sex (male), %	50.6	52.5	0.64
Age (years)	51 ± 11	51 ± 11	0.85
BMI (kg/m^2^)	28.9 ± 3.6	28.5 ± 3.9	0.36
Types of hernia, n (%)			
Primary	387 (86%)	359 (86%)	
Recurrent	35 (7%)	27 (6%)	
Not mentioned	30 (6%)	30 (7%)	
Mean area of defect (cm^2^)	34.52 ± 26.2	34.78 ± 22.4	0.49
Hernia location			
M1	7	3	
M2	57	24	
M3	140	125	
M4	45	49	
M2M3	3	8	
M2M3M4	1	1	
Medial (not subcategorised)	196	82	
Lateral	5	8	
Medial and lateral	0	3	
Types of hernia, n			
Primary hernia	387	359	
Recurrent hernia	35	27	
Not mentioned	30	30	

### Patient and study characteristics

**Fig. 2:**
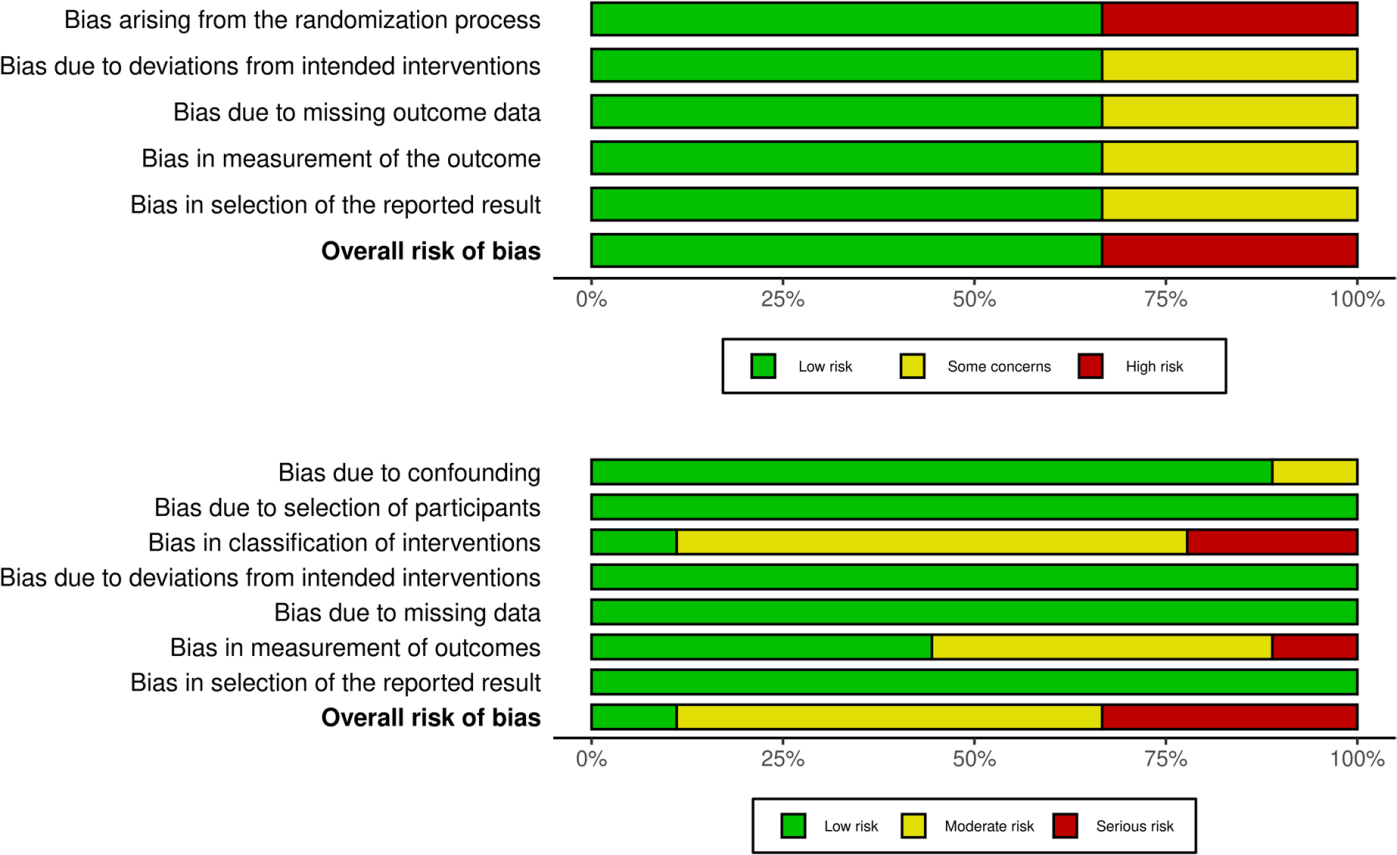
Risk of bias assessment

In total, 868 patients were included in the meta-analysis (452 in IPOM and 416 in eTEP groups). There were 441 males (52.4%,) and the mean (SD) age of the study population was 51 (± 11), years. With regards to the IPOM group, 107 patients underwent laparoscopic IPOM (23.67%), 228 laparoscopic IPOM plus (50.44%) and 117 robotic IPOM plus (25.88%), respectively. In the eTEP group, 297 patients underwent laparoscopic eTEP (71.39%) whilst 119 robotic eTEP (28.60%). Baseline BMI (kgm^2^) of IPOM and eTEP groups were 28.9 and 28.5 respectively. Mean (SD) area of defect (cm^2^) for IPOM and eTEP were 34.52 (± 26.2) and 34.78 (± 22.4) respectively. Follow up duration ranged from 3 months [26, 28] to 6 months [24, 29], and in one study up to 35 months [21]. Further details are provided in Table 2 Fig. 2.

### Postoperative pain at day 7 (POD7)

**Fig. 3:**
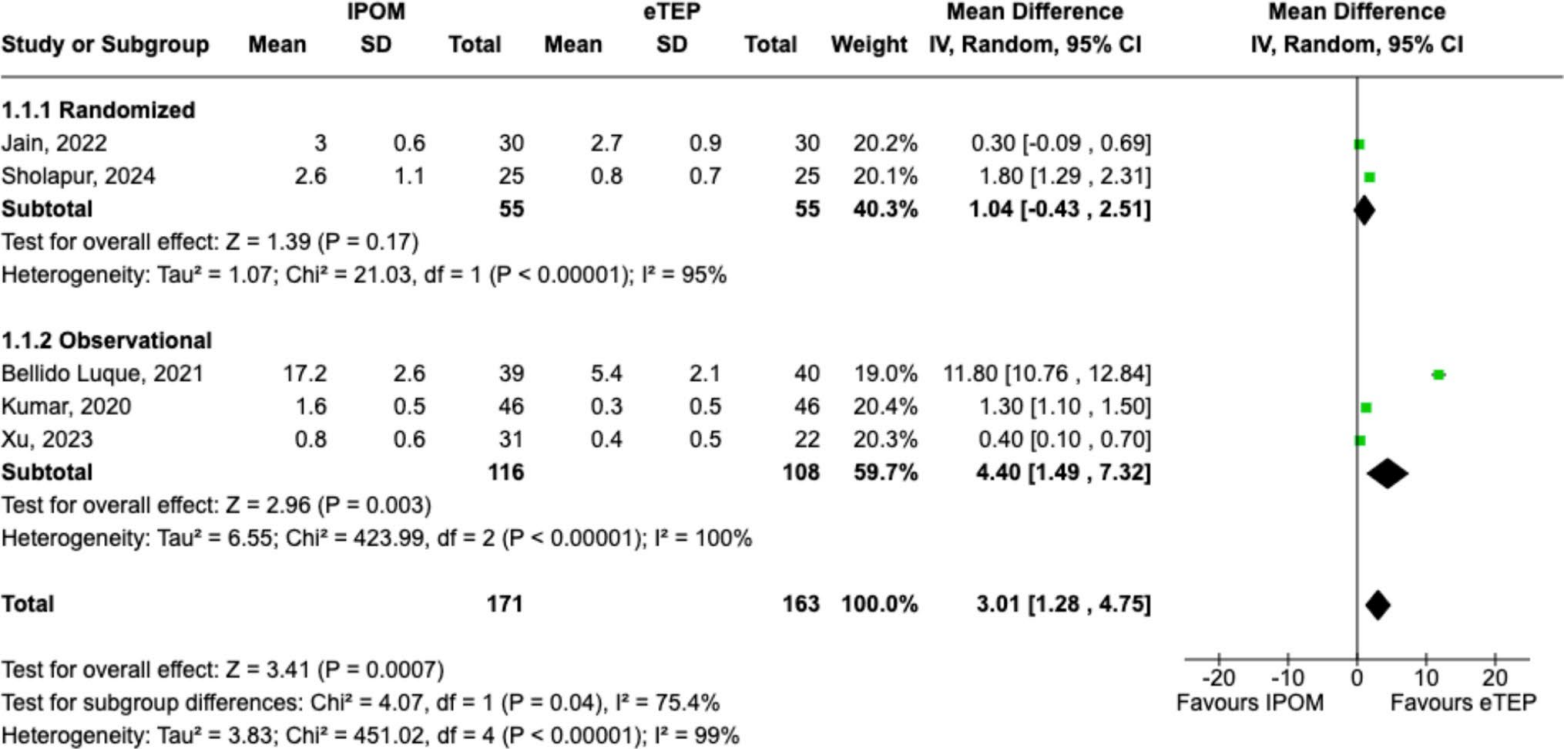
Forest plot comparing pain at POD7 between IPOM and eTEP

IPOM was associated with significantly higher pain scores at POD7 (VAS; MD 3.01, 95%CI = 1.28–4.75, p = 0.0007; Chi^2^ = 4.07, df = 1, p = 0.04, *I*^*2*^ = 75.4%) when compared to eTEP (Fig. 3). However, there was significant statistical heterogeneity noted between groups.

### Operative time

**Fig. 4:**
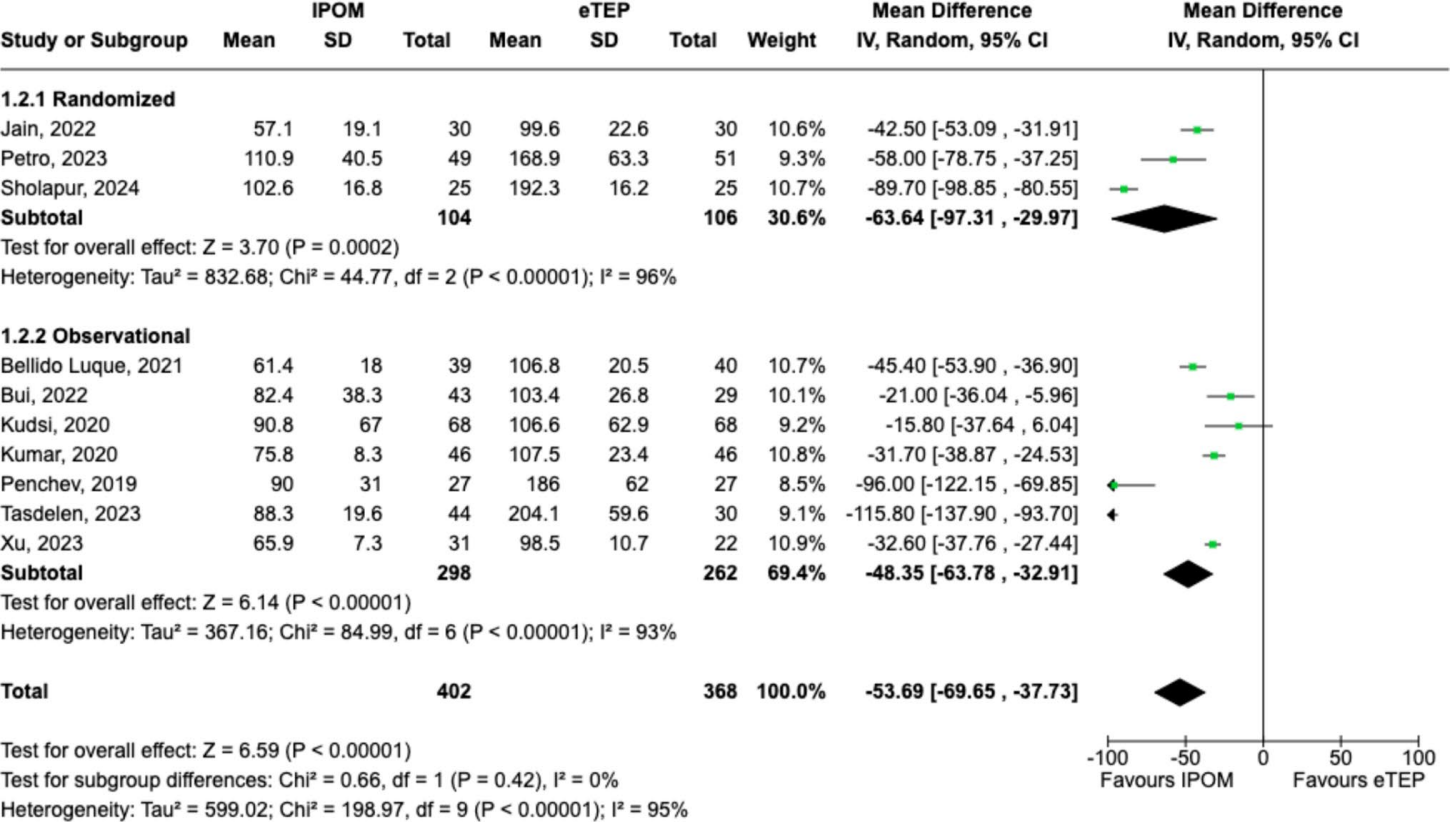
Forest plot comparing operative time between IPOM and eTEP

IPOM was associated with a shorter operative time (MD − 53.69 min, 95%CI = − 69.65- − 37.73, p < 0.00001; Chi^2^ = 0.66, df = 1, p = 0.42, *I*^*2*^ = 0%) when compared to eTEP (Fig. 4).

### Length of stay

**Fig. 5:**
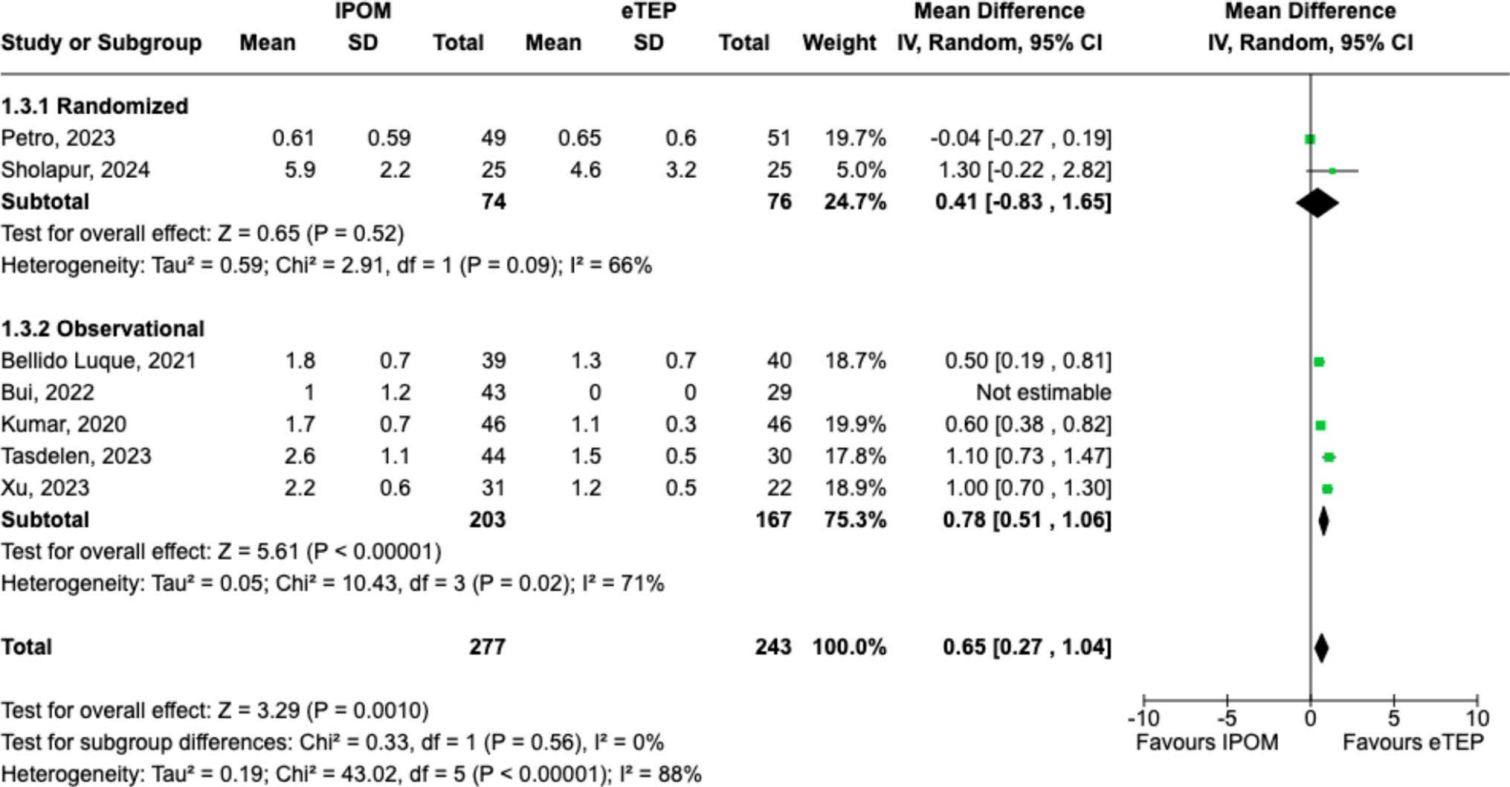
Forest plot comparing length of stay between IPOM and eTEP

IPOM was associated with a longer LOS (MD 0.65 days, 95%CI = 0.27–1.04, p = 0.001; Chi^2^ = 0.33, df = 1, p = 0.56, *I*^*2*^ = 0%) when compared to patients undergoing eTEP (Fig. 5).

### Complications

**Fig. 6:**
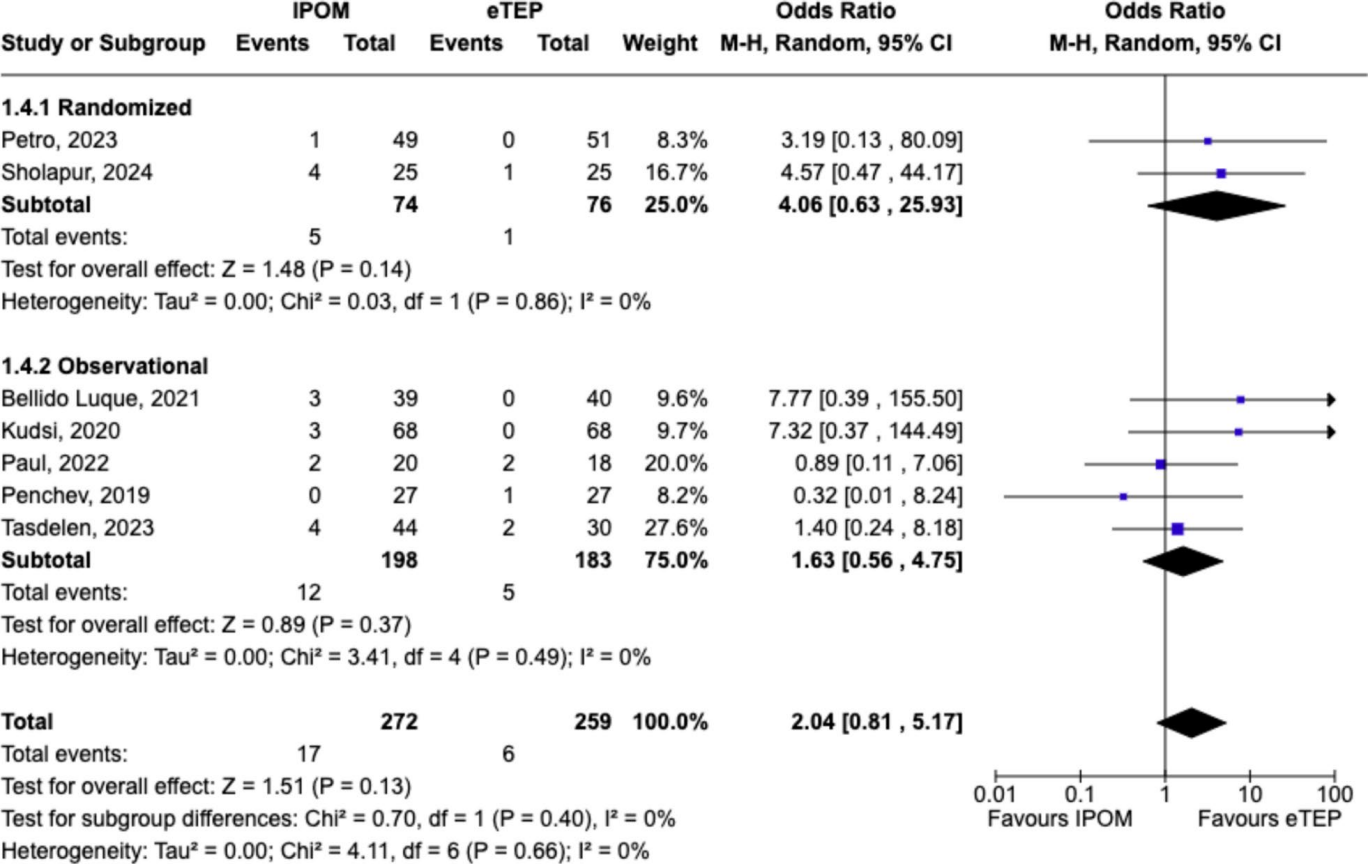
Forest plot comparing intraoperative complications between IPOM and eTEP

In terms of intraoperative complications, there was a higher incidence of bowel injury during IPOM (n = 14, including 3 bowel perforations) when compared to eTEP (n = 3, including 2 bowel perforations). In addition, 3 patients suffered vessel injury during IPOM compared to 2 during eTEP. However, overall, this failed to reach statistical significance (OR 2.04, 95%CI = 0.81–5.17, p = 0.13; Chi^2^ = 0.70, df = 1, p = 0.40, *I*^*2*^ = 0%) (Fig. 6).

When postoperative complications were examined, similarly, there were no statistically significant differences between IPOM and eTEP groups (OR 1.15, 95%CI = 0.54–2.46, p = 0.72; Chi^2^ = 0.29, df = 1, p = 0.59, *I*^*2*^ = 0%) (Fig. 6). Of all postoperative complications, seroma was the commonest in both groups, (40 IPOM versus 45 eTEP). More patients developed paralytic ileus following IPOM (n = 20) when compared to eTEP (n = 3).

### Recurrence

**Fig. 7:**
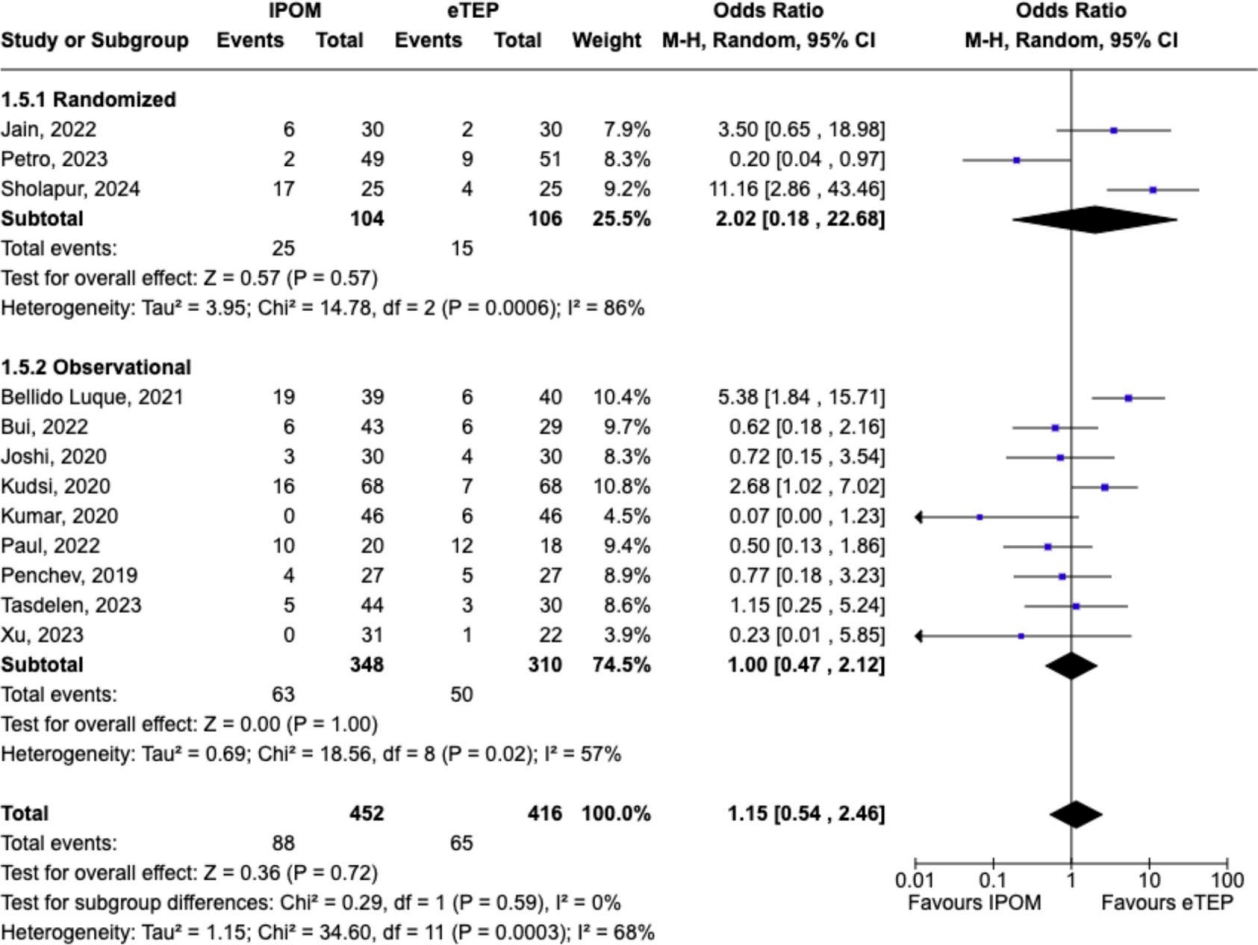
Forest plot comparing postoperative complications between IPOM and eTEP

Overall, no significant differences in recurrence were observed in between groups (OR 2.08, 95%CI = 0.79–5.46, p = 0.14; Chi^2^ = 0.07, df = 1, p = 0.80, *I*^*2*^ = 0%) (Fig. 7).

### Sensitivity analyses

**Fig. 8:**
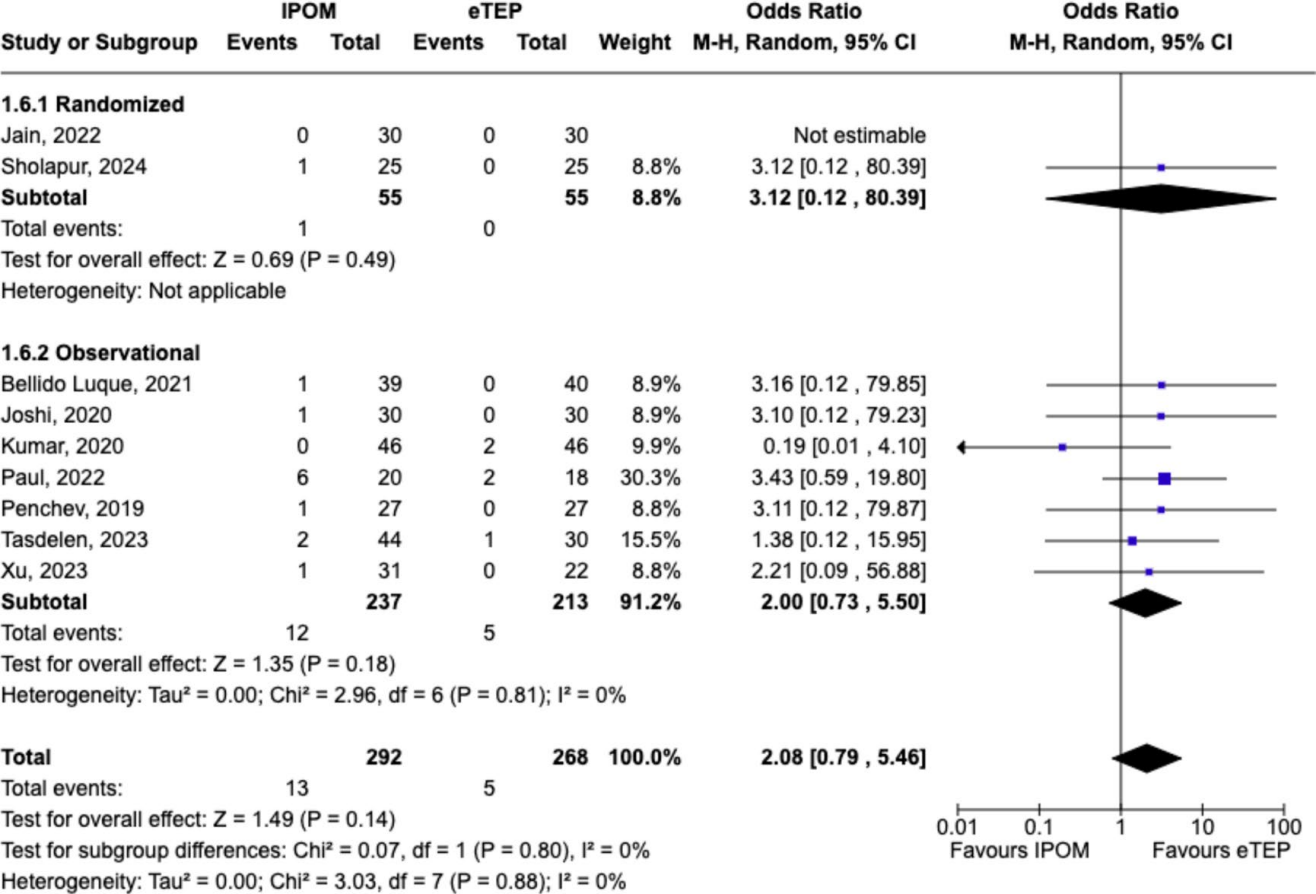
Forest plot comparing recurrence between IPOM and eTEP

A subgroup analysis was carried out, specifically comparing defect closure (laparoscopic IPOM plus) versus laparoscopic eTEP. The results are shown in Table 3 Fig. 8

**Table 3:** Subgroup analysis: laparoscopic IPOM plus (n = 228) vs laparoscopic eTEP (n = 297)

	Lap eTEP vs Lap IPOM plus	
Mean difference [95% CI]		
Pain at POD7 (VAS score)	3.73 [1.52, 5.94]	Chi^2^ = 2.97, df = 1 (P = 0.08), I_2_ = 66.3%
Operative time (min)	− 54.86 [− 76.32, − 33.40]	Chi^2^ = 21.56, df = 1 (P < 0.00001), I^2^ = 95.4%
Length of stay (day)	0.80 [0.53, 1.06]	Chi^2^ = 0.43, df = 1 (P = 0.51), I^2^ = 0%
Odds ratio [95% CI]		
Intraoperative complications	2.74 [0.77, 9.69]	Chi^2^ = 0.28, df = 1 (P = 0.59), I^2^ = 0%
Postoperative complications	1.34 [0.35, 5.14]	Chi^2^ = 6.75, df = 1 (P = 0.009), I^2^ = 85.2%
Recurrence	1.36 [0.36, 5.17]	Chi^2^ = 0.30, df = 1 (P = 0.58), I^2^ = 0%

### Quality assessment and risk of bias

Two of the three RCTs included in this meta-analysis were found to have a low risk of bias across all five domains measured by the ROB 2 tool [23, 24]. The third included RCT was a poorly designed study with a high risk of bias [26]. Only one of nine included non-randomised studies was found to have a low risk of bias when using the ROBINS-I tool [27]. Of the remaining eight non-randomised studies, five were deemed to have moderate risk of bias [20–22, 28, 29] and three serious risk of bias [25, 30, 31]. The low risk Kumar et al*.* study was prospective in nature [27]. Areas of concern identified in the other eight studies included “bias due to confounding”, “bias in classification of intervention” and “bias in measurement of outcome”. Bias due to confounding was an area of concern in one study [23] where pain was not assessed using the VAS score but instead by NRS- 11 score and analgesic requirements. Bias in classification of intervention was an area of concern in eight studies [20–22, 25, 27–29, 31] as there was no description of how patients were allocated to an intervention group. Additionally two studies [30, 31] did not clearly outline the steps of the interventions (IPOM/eTEP). Bias in measurement of outcome was an area of concern in eight studies [20–22, 25, 27–29, 31] as it was unclear in data collectors were blinded to the intervention the patients had undergone.

### Publication bias

This was assessed using a Funnel plot and is shown in Supplementary Fig. 1.

## Discussion

The current systematic review and meta-analysis, encompassing 12 studies and 868 patients, demonstrates significantly more pain at postoperative day 7 as well as longer hospital stay in the IPOM(+) group but no differences in complications or recurrence between the two groups. However, these findings have to be interpreted with caution given that the majority of included studies were observational with a short follow-up (longest duration was 28 months in eTEP group in Bellido Luque et al*.*) [21] and have likely underestimated the true recurrence rate. A robotic (versus a laparoscopic) platform was utilized in two of the studies [23, 28] and the resultant improved ergonomics may have influenced the operative time. Although the majority of hernias operated on across the 12 studies comprised primary ventral hernias, there is now substantial evidence in the literature demonstrating similar safety and efficacy when eTEP is used to repair incisional hernias [32, 33]. It is important to highlight that most hernias were midline hernias in the current meta-analysis and their defects were medium in size at an average of 36 cm^2^. However, eTEP has been shown to be an effective treatment strategy in the repair of subcostal incisional hernias, with lower postoperative pain and shorter hospital stay compared to traditional IPOM +  [33]. Five of the 12 studies described simultaneous TAR with eTEP, performed for bigger defects and/or lateral hernias [22–24, 27, 28] (see Table 1).

Open ventral hernia mesh repair techniques are historically associated with high recurrence rates, ranging from 12 to 52% [34, 35] and hence have been largely superseded by the laparoscopic approach. One of the largest series of laparoscopic IPOM published in 2003, whereby a bridging onlay mesh was inserted without defect closure, demonstrated a recurrence rate of only 4.7% after a mean follow-up of 20.2 months [34]. However, it also had a moderately high postoperative complication rate of 13.2%. Subsequently, Franklin Jr et al., in his series of 384 patients treated with laparoscopic IPOM + with a mean follow-up of 47.1 months, showed that the recurrence rate could be further reduced to 2.9% following defect closure, with an acceptable complication rate (10.1%) [36]. However, defect closure during laparoscopic IPOM is not universally performed as it is technically challenging. In the current meta-analysis, 4 out of 12 studies [24, 29–31] did not close the defect. However, data from all twelve studies were pooled together to generate summative outcomes in the overall analysis. Notwithstanding this, we have performed subgroup analyses comparing IPOM with defect closure (IPOM +) with eTEP and demonstrated that similar findings prevailed, particularly with regards to recurrence (2.7% IPOM + vs. 1.8% eTEP; p = 0.65).

Laparoscopic IPOM, although superior to open repair in terms of faster recovery and return to work and lower wound and mesh complication rates, is not without its pitfalls. Indeed it is associated with poor cosmesis due to skin bulging over the defect, weakness over the bridged gap, mesh eventration as well intraperitoneal bowel adhesions to the mesh [9, 11, 37]. In addition, some patients could experience a certain degree of lack of core support and functional stability when the defect is not closed but simply bridged with a prosthesis [38]. Defect closure, whilst technically cumbersome, may provide a more uniform scaffolding that allows mesh ingrowth and reduce the risk of mesh eventration and skin bulging and seroma formation [37, 39, 40]. However, detractors of defect closure argue that it may convert a tension-free into a tension repair and hence increase the risk of recurrence. A recent meta-analysis by Tryliskyy et al. [41]*.* evaluating IPOM versus IPOM + failed to demonstrate any significant difference between the two techniques when recurrence and major complications were grouped together as a composite outcome. However, the latter study was limited by the inclusion of merely 3 RCTs with divergent hernia and operative characteristics. Further research is warranted to determine if defect closure in IPOM translates into superior patient outcomes.

Enhanced-view extraperitoneal repair (eTEP), on the other hand, involves dissection in the retromuscular/extraperitoneal plane and obviates the need for mesh fixation as the mesh is sandwiched in between the rectus abdominis muscle anteriorly and the rectus sheath posteriorly [37, 42, 43], hence becoming the favoured approach for some surgeons. Due to the more extensive dissection required (as well as the need for transversus abdominis muscle release in cases of large defects), eTEP is associated with longer operative times, as evidenced in the current study. However, no other significant differences were observed between the two techniques. The longer operative times could translate into increased costs but the latter are offset by the use of a simple polypropylene mesh (versus a composite mesh) in cases of eTEP.

Enhanced-view totally extraperitoneal repair requires a sound understanding of abdominal wall anatomy and has a steep learning curve. A 2022 meta-analysis revealed a 2% incidence of intraoperative complications and a 1% incidence of major complications [44]. These include inadvertent puncture of the posterior rectus sheath resulting in pneumoperitoneum, hollow viscus injury, retrorectus haematomas/seromas, damage to epigastric vessels or neurovascular bundles (especially during transversus abdominis release), internal (or interparietal) hernia secondary to dehiscence of the re-approximated posterior sheath, injury to the linea alba during the midline cross-over (resulting in early hernia recurrence) as well as areas of ecchymosis on the abdominal wall (possibly from vascular injury or muscle shearing during anterior sheath closure) [42, 45–47]. With regards to contraindications, eTEP may not be possible in cases with an extensive (from xiphoid to pubis) prior midline incision, as the preperitoneal space would have been violated, rendering the midline cross-over risky if not impossible [47].

Our current analysis has some limitations. Firstly, most of the data originate from observational studies, with their inherent biases as well as short follow-ups. Recurrence rates could be different if patients were followed up for longer. Secondly, whilst most studies employed laparoscopic techniques, two (of twelve) [23, 28] utilized a robotic platform but the data were analysed together in the statistical analysis. Finally, we cannot comment on costs or patient-reported outcome measures (such as core strength and cosmesis) as these were not uniformly reported. Nonetheless, our findings demonstrate that IPOM (+) is as effective as eTEP for ventral hernias in the short-term and both have an acceptable safety profile. It is perhaps prudent to reiterate that ‘not one size fits all’ when it comes to ventral hernias and that some cases may be better served with an IPOM (+) [defect sizes ranging from 2 to 6 cm] and others, eTEP [defect sizes 5–12 cm, ± rectus diastasis] and others, still an open primary/suture repair (< 2 cm defect).

## Supplementary Information

Below is the link to the electronic supplementary material.

Supplementary file1 (DOCX 38 KB)

## Data Availability

All data including spreadsheets are available from the authors upon written request and following agreement on the intended purpose of the request.
