# Supplementary figures and images for: IPOM versus eTEP as minimally invasive approaches for ventral/incisional hernias: a systematic review and meta-analysis
Source: Hernia. 2025 Apr 14;29(1):144. doi: 10.1007/s10029-025-03319-6 (PMC11996944; doi:10.1007/s10029-025-03319-6)

**Supplementary Fig. 1** Funnel plot assessing publication bias


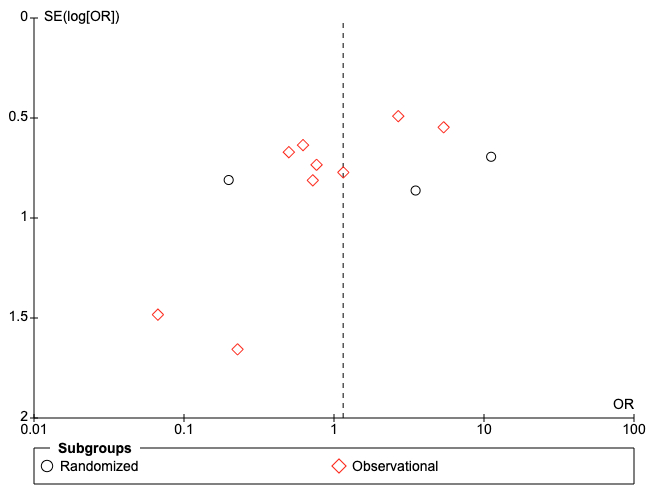

Supplement: Supplementary file 1 — Supplementary file1 (DOCX 38 KB) [file 10029_2025_3319_MOESM1_ESM.docx]
